# Risk of human‐to‐wildlife transmission of SARS‐CoV‐2

**DOI:** 10.1111/mam.12225

**Published:** 2020-10-06

**Authors:** Sophie Gryseels, Luc De Bruyn, Ralf Gyselings, Sébastien Calvignac‐Spencer, Fabian H. Leendertz, Herwig Leirs

**Affiliations:** ^1^ Department of Microbiology, Immunology and Transplantation Rega Institute, KU Leuven Herestraat 49 Leuven 3000 Belgium; ^2^ Department of Ecology and Evolutionary Biology University of Arizona 1041 E. Lowell St. Tucson AZ 85721 USA; ^3^ Department of Biology University of Antwerp Universiteitsplein 1 Antwerp 2610 Belgium; ^4^ Research Institute for Nature and Forest (INBO) Havenlaan 88 Brussels 1000 Belgium; ^5^ Robert Koch Institute Nordufer 20 Berlin 13353 Germany

**Keywords:** COVID‐19, human‐to‐wildlife transmission, mammals, novel reservoir, protective equipment, SARS‐CoV‐2, wildlife

## Abstract

It has been a long time since the world has experienced a pandemic with such a rapid devastating impact as the current COVID‐19 pandemic. The causative agent, severe acute respiratory syndrome coronavirus 2 (SARS‐CoV‐2), is unusual in that it appears capable of infecting many different mammal species. As a significant proportion of people worldwide are infected with SARS‐CoV‐2 and may spread the infection unknowingly before symptoms occur or without any symptoms ever occurring, there is a non‐negligible risk of humans spreading SARS‐CoV‐2 to wildlife, in particular to wild non‐human mammals. Because of SARS‐CoV‐2’s apparent evolutionary origins in bats and reports of humans transmitting the virus to pets and zoo animals, regulations for the prevention of human‐to‐animal transmission have so far focused mostly on these animal groups. We summarise recent studies and reports that show that a wide range of distantly related mammals are likely to be susceptible to SARS‐CoV‐2, and that susceptibility or resistance to the virus is, in general, not predictable, or only predictable to some extent, from phylogenetic proximity to known susceptible or resistant hosts. In the absence of solid evidence on the susceptibility and resistance to SARS‐CoV‐2 for each of the >6500 mammal species, we argue that sanitary precautions should be taken by humans interacting with any other mammal species in the wild. Preventing human‐to‐wildlife SARS‐CoV‐2 transmission is important to protect these animals (some of which are classed as threatened) from disease, but also to avoid establishment of novel SARS‐CoV‐2 reservoirs in wild mammals. The risk of repeated re‐infection of humans from such a wildlife reservoir could severely hamper SARS‐CoV‐2 control efforts. Activities during which direct or indirect interaction with wild mammals may occur include wildlife research, conservation activities, forestry work, pest control, management of feral populations, ecological consultancy work, management of protected areas and natural environments, wildlife tourism and wildlife rehabilitation in animal shelters. During such activities, we recommend sanitary precautions, such as physical distancing, wearing face masks and gloves, and frequent decontamination, which are very similar to regulations currently imposed to prevent transmission among humans. We further recommend active surveillance of domestic and feral animals that could act as SARS‐CoV‐2 intermediate hosts between humans and wild mammals.

## Graphical abstract

SARS‐CoV‐2, the highly transmissible novel coronavirus in humans, has a strikingly broad potential host range. We review the risk and potential impact of human‐to‐wildlife transmission of SARS‐CoV‐2. As demonstrated by several observations of humans transmitting SARS‐CoV‐2 to felids, dogs and mink, and by numerous animal and *in vitro* infection experiments, it is clear the virus is able to infect and be transmitted among a wide range of distantly related mammal species. If SARS‐CoV‐2 was to infect and spread among wild mammals, it could potentially cause disease in some mammal populations, in turn further endangering already threatened species or populations. A new animal reservoir of SARS‐CoV‐2 could be established if the virus were to be sustainably transmitted among some mammalian populations or communities. Such a reservoir could repeatedly source new outbreaks in humans and other animals and would severely hamper control measures of the pandemic. We therefore urge people to take sensible sanitary precautions when in direct or indirect contact with any wild or feral mammal species to prevent human‐to‐wildlife transmission of SARS‐CoV‐2. Controlling transmission routes from humans to wildlife via domestic, commensal or feral mammals acting as intermediate hosts will likely depend on early and active surveillance of these mammals.

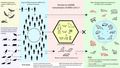

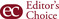

## INTRODUCTION

Humans throughout the world are currently facing one of the most impactful pandemics in history. By 20 August 2020, almost 800000 deaths and 23 million confirmed COVID‐19 cases had been reported worldwide, of which about 6.6 million were people with currently ongoing infection. These numbers are a large underestimation, as many people with mild to moderate symptoms or no symptoms are not tested (Li et al. 2020). A non‐negligible proportion of people can thus be expected to be infected with SARS‐CoV‐2, the coronavirus that causes COVID‐19. This virus is very efficiently transmitted via saliva and nasal droplets, which come into contact with mouth and nose epithelia either directly or indirectly through touching contaminated surfaces, and can travel via exhaled air (Meselson 2020). Infected people are already infectious days before the onset of COVID‐19 symptoms, and some infected people remain asymptomatic yet infectious for several days (Gudbjartsson et al. 2020, Pan et al. 2020, Sutton et al. 2020). While strict social distancing measures have substantially reduced transmission in many areas, complete worldwide eradication is probably not feasible in the near future. SARS‐CoV‐2 is likely to continue to circulate in human populations, probably with oscillations in prevalence, for a considerable time (Kissler et al. 2020).

Transmission of human pathogens to non‐human animals, including wildlife, occurs more regularly than often thought (Epstein & Price 2009, Messenger et al. 2014). SARS‐CoV‐2 appears to have a striking ability to infect a broad range of distantly related mammals. In combination with its high transmissibility and its presence in a significant number of (potentially asymptomatic) people throughout the world, this creates a dangerous situation in which humans may unknowingly transmit the virus to susceptible wild non‐human mammal populations.

We discuss the current evidence for the mammalian evolutionary origins of human coronaviruses, the range of mammals SARS‐CoV‐2 may be able to infect, the potential pathological effects on these mammals, and the likelihood and potential consequences of sustained transmission of SARS‐CoV‐2 among wildlife populations. We summarise precautions that can be taken by people in direct or indirect contact with feral or wild mammals, such as wildlife researchers, conservationists, forestry workers, pest control staff, feral population control staff, ecological consultancy workers, managers and staff of protected areas and natural environments, wildlife tourists, wildlife tourism staff, and staff in wildlife rehabilitation centres. We acknowledge that not only wild and feral mammals, but also captive, domestic and pet mammals can be at risk of acquiring SARS‐CoV‐2 infection from humans. However, in this manuscript, we focus on the prevention and potential consequences of human‐to‐wildlife transmission.

## METHODS

In April 2020, we regularly searched the literature with different combinations of the keywords: ‘SARS‐CoV‐2’, ‘infection experiment’, ‘animal model’, ‘mammal’, ‘susceptibility’, ‘ACE2’, ‘cell line’, ‘coronavirus’, ‘wildlife’, and relevant wildcards, and updated these searches in July and August 2020 during revisions. We particularly checked ProMED (https://promedmail.org/), a community‐driven platform that scans infectious disease news and reports every instance of non‐human animals naturally infected by SARS‐CoV‐2. Many of the SARS‐CoV‐2 studies cited in this perspective have not yet been peer‐reviewed, and have only been described in press releases or in preprint manuscripts, and should thus be interpreted with caution.

## WHAT ARE CORONAVIRUSES AND WHERE DO THEY COME FROM?

Coronaviruses (CoV) are RNA viruses of the family *Coronaviridae*, subfamily *Orthocoronavirinae*, in which four genera can be distinguished: *Alpha*‐, *Beta*‐, *Gamma*‐, and *Deltacoronavirus*. Gamma and delta CoV have been found mostly in avian hosts, while alpha and beta CoV appear to be associated with mammals (Anthony et al. 2017b). Many of the known alpha and beta CoV lineages seem to have a long evolutionary history mostly confined to particular chiropteran genera, though spillovers (i.e. cross‐species transmission events) to other host species, including humans and livestock animals, frequently occur with apparently shorter onwards transmission pathways (Leopardi et al. 2018).

Seven CoVs are known to infect or have infected humans; all seven have an ancestry in other mammalian hosts. Four of these, the alpha HCoV‐NL63 and HCoV‐229E and beta HCoV‐OC43 and HCoV‐HKU1, commonly circulate in people around the world with seasonal oscillations in prevalence, and generally cause mild respiratory symptoms such as the common cold (Su et al. 2016). These viruses are believed to have originated in either bats or rodents (Corman et al. 2018). Nevertheless, closer relatives of HCoV‐229E are found in the alpaca *Vicugna pacos* and the dromedary *Camelus dromedarius*, and the sister clade to HCoV‐OC43 comprises viruses infecting a wide range of mammals, primarily artiodactyls but also canids, suggesting that these virus lineages may have passed a significant proportion of their evolutionary time in non‐bat and non‐rodent hosts before spilling over to humans (Corman et al. 2018, Cui et al. 2019).

The first CoV known to have inflicted severe disease in humans was the severe acute respiratory syndrome coronavirus (SARS‐CoV‐1), which emerged in humans in 2002–2003, with a total of ~8000 confirmed infections and a ~10% case fatality rate. SARS‐CoV‐1 is likely to have had an evolutionary origin in horseshoe bats *Rhinolophus* spp. (Hu et al. 2017), but as this virus was also found to circulate in captive masked palm civets *Paguma larvata* and raccoon dogs *Nyctereutes procyonoides* in markets and some farms, these species may have acted as intermediate hosts (Guan et al. 2003, Kan et al. 2005). It is also possible, however, that rather in reverse, humans acted as an intermediate host for these carnivores.

Middle‐eastern Respiratory Syndrome virus (MERS‐CoV) first emerged in 2012 in humans and has a ~35% case fatality rate. MERS also probably has an evolutionary origin in bats (Vespertilionidae; Ithete et al. 2013, Anthony et al. 2017a), but it is clear that humans repeatedly acquire MERS through close contact with dromedaries without sustained human‐to‐human transmission (Dudas et al. 2018). Dromedaries were probably infected by a MERS ancestor a few decades ago, either directly from bats or via another intermediate host, and are now considered to be the MERS reservoir (Corman et al. 2014).

The novel CoV that emerged in humans in December 2019, SARS‐CoV‐2, is phylogenetically closely related to SARS‐CoV‐1, without being its closest known relative (Zhou et al. 2020a). Rather, each virus forms subclades with CoV mostly found in horseshoe bats (Zhou et al. 2020a). SARS‐CoV‐2 is less deadly than SARS‐CoV‐1 but has a higher transmission rate, further facilitated by asymptomatic and pre‐symptomatic transmission (He et al. 2020). Hence, what started as an epidemic in Wuhan, China, quickly escalated to a pandemic.

As in all CoV, the protein that forms the ‘spikes’ on the virion surface mediates recognition of and entry into host cells. The receptor‐binding domain (RBD) of the spike protein can bind to a particular protein on the surface of host cells, in the case of SARS‐CoV‐2 and many related viruses, the angiotensin‐converting‐enzyme‐2 ACE2 (Wan et al. 2020). Genetic variation in the ACE2 gene among vertebrate species results in variation in chemical properties of the protein. These variations can affect the efficiency with which RBD binds to ACE2, and therefore, ACE2 is a major determinant of a species’ susceptibility to SARS‐CoV‐2 (Wan et al. 2020). Unlike SARS‐CoV‐1, SARS‐CoV‐2 further contains a polybasic cleavage site: an insertion of a few residues that allows host enzymes to cleave the spike protein for more efficient cell entry (Hoffmann et al. 2020).

It is not known which animal species directly infected the first human of the COVID‐19 pandemic, as no CoV similar enough to SARS‐CoV‐2 has yet been found in a non‐human source (Zhang & Holmes 2020). The overall closest relative to SARS‐CoV‐2, strain RATG13, was recovered from an intermediate horseshoe bat *Rhinolophus affinis* (Zhou et al. 2020a). The genome of strain RATG13 is about 96% identical to SARS‐CoV‐2, but, crucially, the gene sequence coding for the RBD differs substantially from that of SARS‐CoV‐2 (Zhou et al. 2020a). The SARS‐CoV‐2 RBD is more closely related to a strain of CoV found in two Malaysian pangolins *Manis javanica* (Lam et al. 2020, Liu et al. 2020a, Zhang et al. 2020a), though that strain forms a sister lineage to the SARS‐CoV‐2‐RATG13 clade in all other gene regions (Lam et al. 2020). However, in some gene regions, two CoV found in *Rhinolophus sinicus* are closer to the SARS‐CoV‐2‐RATG13 lineage, and in other regions, one from *Rhinolophus malayanus* is closer to SARS‐CoV‐2 than RATG13 is (Boni et al. 2020, Zhou et al. 2020b). The pangolin CoV did not contain a polybasic cleavage site, but such a rare insertion was present in the CoV from *Rhinolophus malayanus* (Zhou et al. 2020b).

Mosaic genomes and complicated phylogenetic relationships are not unusual for CoV. They are prone to recombination, where genetic material of different ancestry is exchanged when a host is infected with two distinct CoV strains (Su et al. 2016). A complex history involving several recombination events in natural hosts was also proposed for SARS‐CoV‐1 (Hu et al. 2017). Phylogenetic analyses taking these complex mosaic ancestries into account and using all known SARS‐like CoV, most of which are retrieved from horseshoe bats but a second lineage comes from Malaysian pangolins, suggest that SARS‐CoV‐2 is not the product of recent recombination between known CoV strains (Boni et al. 2020). It is likely to have diverged in its current genomic form from a common ancestor with RATG13 several decades ago (Boni et al. 2020). The subsequent recent stretch of evolutionary history may have taken place solely in the natural reservoir of SARS‐CoV‐2 (possibly a horseshoe bat), or solely or partly in another unidentified natural host (Boni et al. 2020).

## A LARGE NUMBER OF MAMMAL SPECIES ARE AT RISK OF ACQUIRING SARS‐CoV‐2

Various pieces of evidence suggest that SARS‐CoV‐2 is able to infect and be transmitted among many different mammal species. Box 1 provides an overview of the current literature on and the lines of evidence for SARS‐CoV‐2’s potential host range; this literature continues to expand rapidly. In Table 1, we list mammal species for which there is current evidence of susceptibility or resistance to SARS‐CoV‐2 via natural infection observations, animal infection experiments, and *in vitro* infection assays.

**Table 1:** List of mammal species with susceptibility or resistance to SARS‐CoV‐2, based on evidence from natural infection observations, animal infection experiments, and *in vitro* infection assays in which cells of commonly used cell lines carry the ACE2 protein (receptor for SARS‐CoV‐2 entry) of the listed species. Evidence for susceptibility or resistance of a species solely based on *in vitro* assays should be treated with caution (see Box 1). For indirect inference of susceptibility of various other species via ACE2 homology modelling, see Damas et al. (2020), Frank et al. (2020), Liu et al. (2020b), Liu et al. (2020c), Luan et al. (2020), and Melin et al. (2020). vRNA = SARS‐CoV‐2 RNA. Empty cells imply there are no data available for the particular question

Order	Family	Species	Susceptible to SARS‐CoV‐2?	When infected, transmitter of SARS‐CoV‐2?	Does SARS‐CoV‐2 cause disease?	Sources
Artiodactyla	Bovidae	*Bos mutus*	Possibly susceptible^a^			Liu et al. (2020b)
		*Bos taurus*	Possibly susceptible^a^			Liu et al. (2020b)
		*Bubalus bubalis*	Possibly susceptible^a^			Liu et al. (2020b)
		*Capra hircus*	Possibly susceptible^a^			Liu et al. (2020b)
		*Ovis aries*	Possibly susceptible^a^			Liu et al. (2020b)
	Delphinidae	*Globicephala melas*	Possibly susceptible^a^			Liu et al. (2020b)
		*Orcinus orca*	Possibly susceptible^a^			Liu et al. (2020b)
		*Tursiops truncatus*	Possibly susceptible^a^			Liu et al. (2020b)
	Lipotidae	*Lipotes vexillifer*	Possibly susceptible^a^			Liu et al. (2020b)
	Monodontidae	*Delphinapterus leucas*	Possibly susceptible^a^			Liu et al. (2020b)
	Phocoenidae	*Neophocaena asiaeorientalis asiaeorientalis*	Possibly susceptible^a^			Liu et al. (2020b)
	Physeteridae	*Physeter catodon*	Probably not susceptible^b^			Liu et al. (2020b)
	Suidae	*Sus scrofa*	Probably not susceptible. *In vitro* studies show cellular infection could be possible, but no virus replication or seroconversion was in observed in experimentally infected real animals	Unlikely to transmit virus; no excreta positive for vRNA		Liu et al. (2020b), Schlottau et al. (2020), Shi et al. (2020), Zhou et al. (2020a)
Carnivora	Canidae	*Canis lupus familiaris*	Susceptible, but no efficient replication in some breeds	No transmission to conspecifics observed in infection experiment. Some excreta samples positive for vRNA; if these represent infectious virus, transmission could be possible	Mild to severe illness observed in some	AFCD (2020b), Liu et al. (2020b), MANFQ (2020c), Shi et al. (2020), Sit et al. (2020), USDA (2020), Zhao et al. (2020b)
		*Nyctereutes procyonoides*	Susceptible, but infection failed in 3/9 experimentally infected animals	Efficient transmission observed in animal experiment	No observed illness	Freuling et al. (2020), Zhao et al. (2020b)
		*Vulpes vulpes*	Probably susceptible			Liu et al. (2020b)
	Felidae	*Felis catus*	Susceptible	Efficient transmission observed in animal experiments, with evidence for airborne transmission	Mild to severe illness occurs in some	AFCD (2020a, 2020b), Bosco‐Lauth et al. (2020), FASFC (2020), Halfmann et al. (2020), Kim et al. (2020), Liu et al. (2020b), Newman et al. (2020), OIE (2020a, Oreshkova et al. (2020), 2020b), Sailleau et al. (2020), Shi et al. (2020), Temmam et al. (2020), USDA (2020), Zhang et al. (2020b), Zhao et al. (2020b)
		*Lynx canadensis*	Probably susceptible^c^			Liu et al. (2020b)
		*Panthera leo*	Susceptible	Excreta samples positive for vRNA; if these represent infectious virus, transmission could be possible	Mild illness occurred in some	Liu et al. (2020b), McAloose et al. (2020)
		*Panthera pardus*	Probably susceptible^c^			Liu et al. (2020b)
		*Panthera tigris*	Susceptible	Excreta samples positive for vRNA; if these represent infectious virus, transmission could be possible	Mild illness occurred in some	Liu et al. (2020b), McAloose et al. (2020)
		*Puma concolor*	Probably susceptible^c^			Liu et al. (2020b)
	Mustelidae	*Arctonyx collaris*	Probably susceptible^c^			Zhao et al. (2020b)
		*Melogale moschata*	Probably susceptible^c^			Zhao et al. (2020b)
		*Mustela erminea*	Probably susceptible^c^			Liu et al. (2020b)
		*Mustela putorius furo*	Susceptible	Efficient transmission observed in animal experiments, including airborne		Kim et al. (2020), Richard et al. (2020), Schlottau et al. (2020), Shi et al. (2020)
		*Neovison neovison*	Susceptible	Efficient transmission observed in farms, including to humans and probably airborne	Variation in illness: from asymptomatic or mild disease to severe illness and death	MANFQ (2020a, 2020b), MEFD (2020), Oreshkova et al. (2020), USDA (2020)
	Otariidae	*Eumetopias jubatus*	Possibly susceptible^a^			Liu et al. (2020b)
		*Zalophus californianus*	Possibly susceptible^a^			Liu et al. (2020b)
	Phocidae	*Neomonachus schauinslandi*	Possibly susceptible^a^			Liu et al. (2020b)
	Ursidae	*Ailuropoda melanoleuca*	Possibly susceptible^a^			Liu et al. (2020b)
		*Ursus arctos horribilis*	Possibly susceptible^a^			Liu et al. (2020b)
	Viverridae	*Paguma larvata*	Possibly susceptible^a^			Zhao et al. (2020b), Zhou et al. (2020a)
Chiroptera	Molossidae	*Tadarida brasiliensis*	Possibly susceptible^a^			Zhao et al. (2020b)
	Pteropodidae	*Rousettus aegyptiacus*	Susceptible	Transmission to 1 out of 3 co‐housed animals observed in one experiment	No observed illness	Liu et al. (2020b), Schlottau et al. (2020)
	Rhinolophidae	*Rhinolophus alcyone*	Possibly susceptible^a^			Hoffmann et al. (2020)
		*Rhinolophus sinicus*	Possibly susceptible^a^			Zhao et al. (2020b), Zhou et al. (2020a)
Diprotodontia	Phascolarctidae	*Phascolarctos cinereus*	Possibly susceptible^a^			Liu et al. (2020b)
Lagomorpha	Leporidae	*Oryctolagus cuniculus*	Possibly susceptible^a^			Liu et al. (2020b), Zhao et al. (2020a)
Perissodactyla	Equidae	*Equus caballus*	Possibly susceptible^a^			Liu et al. (2020b)
	Rhinocerotidae	*Ceratotherium simum simum*	Possibly susceptible^a^			Liu et al. (2020b)
Pholidota	Manidae	*Manis javanica*	Possibly susceptible^a^			Liu et al. (2020b), Zhao et al. (2020b)
Primates	Cebidae	*Callithrix jacchus*	Susceptible, but seemingly less efficient viral replication than in Old World primates			Lu et al. (2020), Liu et al. (2020b)
		*Saimiri boliviensis boliviensis*	Probably susceptible^b^			Liu et al. (2020b)
		*Sapajus apella*	Probably not susceptible			Liu et al. (2020b)
	Cercopithecidae	*Chlorocebus aethiops*	Susceptible		Mild to severe illness	Woolsey et al. (2020)
		*Macaca fascicularis*	Susceptible		Mild to severe illness	Deng et al. (2020), Munster et al. (2020), Rockx et al. (2020), Shan et al. (2020), Yu et al. (2020), Y. Liu et al. (2020b)
		*Macaca mulatta*	Susceptible		Mild to severe illness	Deng et al. (2020), Munster et al. (2020), Rockx et al. (2020), Shan et al. (2020), Yu et al. (2020), Zhao et al. (2020b)
		*Papio anubis*	Probably susceptible^c^			Liu et al. (2020b)
		*Piliocolobus tephrosceles*	Probably susceptible^c^			Liu et al. (2020b)
		*Rhinopithecus roxellana*	Probably susceptible^c^			Liu et al. (2020b)
		*Theropithecus gelada*	Probably susceptible^c^			Liu et al. (2020b)
	Hominidae	*Gorilla gorilla gorilla*	Probably susceptible^c^			Liu et al. (2020b)
		*Homo sapiens*	Susceptible	Efficient transmission, including airborne	Strong variation in illness: from asymptomatic or mild disease to severe illness and death	https://www.worldometers.info/coronavirus/, Hoffmann et al. (2020), Liu et al. (2020b), Zhao et al. (2020b), Zhou et al. (2020a)
		*Pan troglodytes*	Probably susceptible^c^			Liu et al. (2020b)
		*Pongo abelii*	Probably susceptible^c^			Liu et al. (2020b)
	Hylobatidae	*Nomascus leucogenys*	Probably susceptible^c^			Liu et al. (2020b)
Rodentia	Cricetidae	*Cricetulus griseus*	Probably susceptible^c^			Liu et al. (2020b)
		*Mesocricetus auratus*	Susceptible	Efficient transmission observed in animal experiments, including airborne	Mild illness	Chan et al. (2020), Sia et al. (2020)
		*Peromyscus leucopus*	Probably susceptible^c^			Liu et al. (2020b)
		*Peromyscus maniculatus*	Susceptible	Efficient transmission observed in animal experiments	Mild illness	Griffin et al. (2020)
	Dipodidae	*Jaculus jaculus*	Possibly susceptible^a^			Liu et al. (2020b)
	Muridae	*Mus musculus domesticus*	Not susceptible. However, a single amino‐acid substitution in SARS‐CoV‐2 spike protein (though not observed among human isolates) can render laboratory mice susceptible			Bao et al. (2020), Gu et al. (2020), Zhao et al. (2020b), Zhou et al. (2020a)
		*Rattus norvegicus*	Probably not susceptible^b^			Zhao et al. (2020b)
	Sciuridae	*Ictidomys tridecemlineatus*	Possibly susceptible^a^			Liu et al. (2020b)
Scandentia	Tupaiidae	*Tupaia belangeris*	Susceptible		Mild illness in some	Zhao et al. (2020a)

^a^ Possibly susceptible: SARS‐CoV‐2 can enter cells with this species' receptor (ACE2). No natural or experimental infection data of species with similar enough SARS‐CoV‐2 receptor gene (ACE2 gene) are available.

^b^ Probably not susceptible: SARS‐CoV‐2 could not enter cells with this species' receptor (ACE2).

^c^ Probably susceptible: SARS‐CoV‐2 can enter cells with this species' receptor (ACE2). Natural or experimental infection data show SARS‐CoV‐2 can infect other related species with similar enough SARS‐CoV‐2 receptor gene.

**Which wild mammals are, and are not, susceptible to SARS‐CoV‐2?**

Here, we provide an overview of the current, and rapidly expanding, pieces of evidence that show that many different distantly related non‐human wild mammals are susceptible to SARS‐CoV‐2, while others appear to be resistant to the virus. We can classify current evidence of SARS‐CoV‐2 susceptibility into four categories: infections that occurred naturally, animal infection experiments, *in vitro* infection experiments with cell lines, and *in silico* 3D structure modelling of ACE2‐spike protein interactions.

**Natural infections**

In mid‐March 2020, a veterinary diagnostics company tested for the presence of SARS‐CoV‐2 in 4000 pets in the USA. None was positive, but this testing occurred at a time when there were only about 3000 confirmed human cases in the USA (IDEXX 2020). Two asymptomatic pet dogs *Canis familiaris* in Hong Kong were found to be SARS‐CoV‐2‐positive during a small investigation testing 17 dogs and eight cats *Felis catus* from households with confirmed COVID‐19 cases or close contacts of such cases (AFCD 2020b, Sit et al. 2020), and a further fifteen pet dogs with mild to severe symptoms whose owners were confirmed to have COVID‐19 were detected in the USA and one in the Netherlands (MANFQ 2020c, USDA 2020). Twenty‐five pet cats (15 in the USA, two in Belgium, one in Hong Kong, two in France, two in Spain, one in Germany, one in the UK, and one in Russia) with COVID‐19‐positive owners were confirmed to have ongoing or past SARS‐CoV‐2 infection (AFCD 2020a, 2020b, FASFC 2020, Newman et al. 2020, OIE 2020a, 2020b, Sailleau et al. 2020, USDA 2020). These cats mostly displayed mild respiratory symptoms, though some also had digestive problems or fever. On the other hand, in a student community where 13 out of 20 people probably had COVID‐19, none of the nine cats and 12 dogs living with the students was virus‐positive or displayed anti‐SARS‐CoV antibodies (Temmam et al. 2020). In the city of Wuhan, China, where the epidemic is likely to have started, 102 cats were tested for the presence of neutralising anti‐SARS‐CoV‐2 antibodies, of which 15% were positive (Zhang et al. 2020b). These serological tests indicate a past infection from which the cats recovered. Thirty‐nine cats sampled before the start of the outbreak (i.e. before December 2019) were all negative.

Five co‐housed tigers *Panthera tigris* and three co‐housed lions *Panthera leo* in the Bronx Zoo, New York, USA, acquired SARS‐CoV‐2 infection from COVID‐19‐positive members of staff (McAloose et al. 2020). Transmission from staff to the tigers occurred independently from transmission from staff to the lions, but it is not known whether this was indirect or direct, nor whether transmission occurred among the animals as well (McAloose et al. 2020).

So far, SARS‐CoV‐2 has spread among American mink *Neovison vison* individuals in 35 different fur farms in the Netherlands, four in Denmark, two in the United States, and one in Spain. For several outbreaks, the initial infections were confirmed to have been transmitted from staff with COVID‐19 symptoms (MANFQ 2020a, 2020b, MEFD 2020, Oreshkova et al. 2020, USDA 2020). The animals showed either no symptoms or a range of gastro‐intestinal and respiratory symptoms, and an increased mortality rate was noted in some of the farms (Molenaar et al. 2020). Seven out of 24 stray cats found in the surroundings of two of the infected mink farms in the Netherlands were found to carry antibodies against SARS‐CoV‐2 (Oreshkova et al. 2020). These cats were probably infected by the mink, either each individually or via cat‐to‐cat transmission, as they roam around the farms but reportedly do not enter people’s houses. Two instances of subsequent mink‐to‐human transmission were further documented in people that worked at two of the Dutch farms (Oreshkova et al. 2020). All the infected mink farms in the Netherlands are located close together in two provinces, and for several, the route of initial infection is unknown (MANFQ 2020b). This raises the possibility of transmission via undetected non‐human intermediate hosts present in the area, though undetected human infections or other undetected human‐mediated routes are also possible.

**Animal infection experiments**

SARS‐CoV‐2 does not replicate in intranasally inoculated outbred laboratory mice unless they are genetically modified to express human ACE2 (Bao et al. 2020). This suggests that wild house mice *Mus musculus domesticus* are not susceptible to the virus, but the question requires further investigation, especially since only a single amino‐acid substitution in SARS‐CoV‐2’s RBD (which evolved after only six passages) renders BALB/c mice (a commonly used inbred laboratory house mouse strain) susceptible (Gu et al. 2020). Syrian hamsters *Mesocricetus auratus* and the North American deer mouse *Peromyscus maniculatus*, both rodents of the Cricetidae family, are susceptible and readily transmitted the virus to co‐housed conspecifics (Chan et al. 2020, Griffin et al. 2020, Sia et al. 2020). Pigs *Sus scrofa* do not appear susceptible, with no virus replication after experimental inoculation (Schlottau et al. 2020, Shi et al. 2020). SARS‐CoV‐2 does replicate in all tested carnivores: domestic dogs *Canis familiaris*, ferrets *Mustela putorius furo*, raccoon dogs *Nyctereutes procyonoides,* and domestic cats *Felis catus* (Bosco‐Lauth et al. 2020, Freuling et al. 2020, Halfmann et al. 2020, Kim et al. 2020, Richard et al. 2020, Schlottau et al. 2020, Shi et al. 2020). In contrast to in cats, raccoon dogs, and ferrets, replication and viral shedding are low in dogs, and transmission to co‐housed naïve dogs does not occur (Shi et al. 2020). Shi et al. (2020) found that the infected cats transmitted the virus to two out of six non‐inoculated cats, in one instance with evidence for airborne transmission; Halfmann et al. (2020) found transmission in three out of three cat co‐housing situations. Infection of ferrets and efficient ferret‐to‐ferret transmission, including some airborne, was demonstrated in several studies (Kim et al. 2020, Richard et al. 2020, Schlottau et al. 2020, Shi et al. 2020). Rhesus macaques *Macaca mulatta,* crab‐eating macaques *Macaca fascicularis,* and African green monkeys *Chlorocebus aethiops*, often used as non‐human primate model species in biomedical research, are also permissive to infection and develop similar viral shedding dynamics and symptoms as human COVID‐19 patients, with more severe symptoms in *Macaca mulatta* than in *Macaca fascicularis* (Deng et al. 2020, Lu et al. 2020, Munster et al. 2020, Rockx et al. 2020, Shan et al. 2020, Woolsey et al. 2020, Yu et al. 2020). In contrast, SARS‐CoV‐2 genetic material is detectable in the blood and excreta of intranasally inoculated common marmosets *Callithrix jacchus*, but not in organs, and marmosets remain visibly asymptomatic (Lu et al. 2020). SARS‐CoV‐2 is able to replicate in some, but not all, intranasally inoculated tree shrews *Tupaia belangeris*, another emerging laboratory model species (Zhao et al. 2020a). Fever was the only clinical sign some individuals displayed (Zhao et al. 2020a). Nasally inoculated Egyptian fruit bats *Rousettus* *aegypticus* were permissive to infection but showed no clinical symptoms, and the infection was transmitted to one out of three co‐housed individuals (Schlottau et al. 2020). SARS‐CoV‐2 did not replicate in any of the birds that were experimentally inoculated: domestic ducks *Anas platyrhynchos domesticus*, chickens *Gallus gallus*, turkeys *Meleagris gallopavo f. domestica*, Japanese quail *Coturnix japonica,* and white Chinese geese *Anser cygnoides* (Schlottau et al. 2020, Shi et al. 2020, Suarez et al. 2020).

**In vitro *cell culture infection experiments***

The ability of a virus to infect cultured cells *in vitro* provides evidence for its ability to infect tissues of the animal species from which these cells are derived. This does not necessarily mean that the virus would be able to replicate efficiently enough in the actual bodies of these animal species, or that transmission to other individuals would be possible.

For most infection experiments with laboratory cell lines mentioned below, an engineered chimera of a standard laboratory virus strain in which (part of) the SARS‐CoV‐2 spike protein was inserted is used, as this method has been shown to model infection with the real virus into cells quite well (Becker et al. 2008, Letko et al. 2020). Many cell culture lines, however, do not (sufficiently) express the ACE2 proteins that SARS‐like viruses need to bind with to initiate infection. This explains, for example, why SARS‐CoV‐1 and SARS‐CoV‐2 failed to infect the RhiLu/1.1 cell line derived from the halcyon horseshoe bat *Rhinolophus alcyone* (Letko et al. 2020), while human‐derived HeLa cells transfected with *Rhinolophus alcyone* ACE2 protein were successfully infected with SARS‐CoV‐2 (Hoffmann et al. 2020). *In vitro* studies mostly rely on modifying commonly used cell lines to express ACE2 proteins (derived from several species), so they only reflect the ability of SARS‐CoV‐2 to enter cells with ACE2 protein of a particular species, and do not necessarily show whether or not intracellular replication would occur in that species.

We found four studies in which HeLa or BHK‐21 cells were modified to express the ACE2 protein of a total of 59 different mammal species, and in 50 of them, SARS‐CoV‐2 was able to infect these cells (Hoffmann et al. 2020, Liu et al. 2020b, Zhao et al. 2020b, Zhou et al. 2020a). This proportion is probably biased, as in the (not yet peer‐reviewed) study in which 49 of the 59 species were investigated, species were selected for which the ACE2 sequence was already estimated to be able to bind to SARS‐CoV‐2’s RBD, based on structure homology models (Liu et al. 2020b). Nevertheless, as this large number of potentially susceptible mammal species is scattered across the mammalian phylogeny, including, for example Chinese horseshoe bats *Rhinolophus sinicus*, white‐footed mice *Peromyscus leucopus*, belugas *Delphinapterus leucas*, giant pandas *Ailuropoda melanoleuca,* and white rhinoceros *Ceratotherium simum*, these *in vitro* studies further support the conclusion that SARS‐CoV‐2 is able to infect a wide range of distantly related mammals. SARS‐CoV‐2 was able to infect cells transfected with ACE2 of domestic pigs and in a kidney pig cell line (Chu et al. 2020), but apparently not in experimentally inoculated live pigs (Shi et al. 2020; see above), showing a discrepancy between inference from *in vitro* and *in vivo* studies. HeLa cells transfected with *Rhinolophus sinicus* ACE2 were infectable, while lung and kidney cell lines of *Rhinolophus sinicus* were not permissive to SARS‐CoV‐2 infection, despite the fact that the kidney cells were permissive to SARS‐CoV‐1 infection, suggesting that ACE2 was indeed expressed on the cell surface (Chu et al. 2020). This discrepancy could perhaps also be explained by use of *Rhinolophus sinicus* material from different geographical sources, as distinct *Rhinolophus sinicus* populations apparently differ substantially in their ACE2 gene sequence (Frank et al. 2020, Guo et al. 2020). In short, *in vitro* infection studies should be interpreted with care.

**In silico *3D structure modelling of ACE2‐spike protein interactions***

The 3D structure of ACE2 homologs of different vertebrate species can be modelled, based on comparison with the known human ACE2 structure. Theoretically, one can then infer whether or not the RBD of SARS‐CoV‐2 would be able to bind to that species’ ACE2 protein, if its sequence is available. The extent to which we can subsequently infer whether a species is susceptible to SARS‐CoV‐2, let alone the possibility of transmission between actual bodies of the species, is still largely unknown. The uncertainty increases with increasing divergence from the human ACE2 sequence (of which the 3D structure in complex with SARS‐CoV‐2 has actually been determined [Frank et al. 2020, Lan et al. 2020, Shang et al. 2020, Yan et al. 2020]). The binding capacity of ACE2 with SARS‐CoV‐2 does not necessarily decrease with more divergence from humans. In the case of SARS‐CoV‐1, single residue changes in ACE2 can alter the binding efficiency with SARS‐CoV‐1’s RBD significantly (W. Li et al. 2005). The many manuscripts currently appearing in which such species’ susceptibility predictions are based solely on ACE2 sequence analyses should thus be interpreted with caution.

Still, the main conclusion from *in silico* ACE2‐RBD binding studies is in line with evidence from natural and experimental infections: a wide range of mammals may be susceptible, and phylogenetic proximity to a known susceptible host has limited value as a predictor of a species’ susceptibility. Almost all catarrhine primates (apes and Old World monkeys) have identical residues at the sites thought to interact with SARS‐CoV‐2’s RBD, making it likely that all these species are susceptible to SARS‐CoV‐2 (Damas et al. 2020, Frank et al. 2020, Melin et al. 2020). The ACE2 in New World monkeys contains some key differences that might lead to resistance to the virus, according to structure models (Damas et al. 2020, Frank et al. 2020, Melin et al. 2020). However, as mentioned above, SARS‐CoV‐2 replication occurred in at least one species of New World monkey after experimental infection (in a not‐yet peer reviewed study), though with rapid clearance and no disease symptoms (Lu et al. 2020). Structure models further also indicate that mammals with inferred binding compatibility with SARS‐CoV‐2’s RBD are scattered across the mammalian phylogenetic tree (Damas et al. 2020, Frank et al. 2020, Liu et al. 2020b, 2020c, Luan et al. 2020, Melin et al. 2020). Unpredictability from phylogenetic proximity could be especially the case among Chiropteran species, as the available data so far show that the genetic diversity of ACE2, and in particular of the residues implicated in contacting SARS‐CoV’s RBDs, is substantially greater among bat species than among species in other mammalian orders (Hou et al. 2010, Frank et al. 2020, Melin et al. 2020). Therefore, despite the likely evolutionary ancestry of SARS‐like viruses in horseshoe bats, the disproportionally large chiropteran ACE2 diversity suggests that SARS‐CoV‐2 susceptibility may perhaps vary much more among bat taxa than among clades of other mammalian orders.

Natural observations and infection experiments unequivocally show that SARS‐CoV‐2 is able to infect and be transmitted among at least domestic cats *Felis catus*, ferrets *Mustela putorius furo*, American mink *Neovison vison*, raccoon dogs *Nyctereutes procyonoides*, Egyptian fruit bats *Rousettus aegypticus*, North American deer mice *Peromyscus maniculatus,* and Syrian hamsters *Mesocricetus auratus* (Griffin et al. 2020, Halfmann et al. 2020, Kim et al. 2020, Oreshkova et al. 2020, Richard et al. 2020, Schlottau et al. 2020, Shi et al. 2020, Sia et al. 2020). Tigers *Panthera tigris,* lions *Panthera leo,* and macaques *Macaca fascicularis* and *Macaca mulatta* are susceptible, and transmission within these species is undocumented, but likely (Deng et al. 2020, McAloose et al. 2020, Munster et al. 2020). Domestic dogs *Canis familiaris*, tree shrews *Tupaia belangeris,* and common marmosets *Callithrix jacchus* also appear to be susceptible, but appear less likely to able to transmit the virus sustainably onwards (Lu et al. 2020, Shi et al. 2020, Zhao et al. 2020a). Indirect evidence based on *in vitro* assays and *in silico* host‐cell‐receptor binding modelling further show that most Old World primates are likely to be susceptible, as well a high number of distantly related mammal species, with several examples from almost every mammalian order (Damas et al. 2020, Frank et al. 2020, Hoffmann et al. 2020, Luan et al. 2020, Liu et al. 2020b, 2020c). Several species that were predicted not to be susceptible via these *in vitro* and *in silico* analyses belong to the orders or families of mammals that include known SARS‐CoV‐2 susceptible species. For example, most New World primates may not be susceptible, and while Cricetidae rodents such as hamsters and *Peromyscus* spp. may be susceptible, house mice *Mus musculus* and Norway rats *Rattus norvegicus* of the Muridae family appear not to be (Bao et al. 2020, Damas et al. 2020, Griffin et al. 2020, Liu et al. 2020b, Melin et al. 2020, Sia et al. 2020, Zhao et al. 2020b). Despite the probable evolutionary origins of SARS‐CoV‐2 and related viruses in *Rhinolophus* bats, *in vitro* and *in silico* studies suggest that several horseshoe bat species are not susceptible to SARS‐CoV‐2 (Chu et al. 2020, Frank et al. 2020). Based on the above‐average genetic variation in the ACE2 gene among bat species, inter‐species variation in SARS‐CoV‐2 susceptibility might be particularly high among bats in general (Frank et al. 2020). This further complicates attempts to predict which bat species might be susceptible.

SARS‐CoV‐2 is thus able to infect a wide range of mammal species, some of which are only distantly related to each other, and it is not possible to predict the susceptibility of a species based only on its phylogenetic proximity to, for example, humans, horseshoe bats, cats or pangolins. As it is impossible to determine susceptibility individually for all ~6500 known mammal species (Burgin et al. 2018), people interacting directly or indirectly with any wild mammal species should take sanitary precautions to prevent SARS‐CoV‐2 transmission to wildlife.

## ROUTES OF HUMAN‐TO‐WILDLIFE TRANSMISSION

Though most people very rarely come into close contact with live wild animals, transmission of SARS‐CoV‐2 from humans could readily occur during (field) activities of wildlife researchers, conservationists, forestry workers, pest control staff, feral population control staff, ecological consultancy workers, managers and staff of protected areas and natural environments, wildlife tourists, wildlife tourism staff, and staff in wildlife rehabilitation centres. Any situation in which direct contact occurs, in which it is not possible to maintain at least 1 m distance between an infected human and a SARS‐CoV‐2‐susceptible mammal, or where human‐contaminated material may come into contact with susceptible mammals, has a considerable risk of human‐to‐animal transmission. Nevertheless, activities with wild mammals do not necessarily have to be suspended during the COVID‐19 pandemic, as long as relatively straightforward sanitary precautions are taken. In Box 2, we provide an overview of sanitary measures that should be taken by people interacting directly and indirectly with wild mammals, such as field researchers, and how these may differ from standard field biosafety procedures. These measures are in line with recommendations posed by several governmental wildlife agencies (e.g. the U.S. Fish and Wildlife Service) and bat organisations (e.g. the International Union for Conservation of Nature [IUCN] Bat Specialist Group https://www.iucnbsg.org/, EUROBATS https://www.eurobats.org/node/2602), and are in fact largely the same as those imposed within human populations (see https://www.who.int/emergencies/diseases/novel‐coronavirus‐2019/advice‐for‐public/): practice physical distancing and decontaminate surfaces that other animals may come in contact with. If physical distancing is not possible, wear a face mask and decontaminate surfaces in direct or indirect contact with one’s body. The precautions and protective equipment we recommend are not necessarily the same as those used when protecting oneself against a variety of infections carried by wild animals. We recommend following the sanitary guidelines when undertaking all activities with any mammal species, until more evidence is available about which mammalian taxa are resistant to SARS‐CoV‐2 infection.

**Guidelines to prevent SARS‐CoV‐2 transmission to wildlife**

Many fieldworkers interacting with live or dead wild animals are accustomed to taking protective measures to protect themselves from animal infections. For example, biologists may be used to handling rodents in hantavirus endemic areas (Kelt et al. 2010) or preventing cross‐contaminating infections between individual mammals or populations (e.g. when working with bats in areas affected by white‐nose syndrome; White‐Nose Response Team 2018). Preventing the transmission of one’s own viral infections to mammals requires a somewhat different set of precautions. Many other fieldworkers have little or no experience with biosafety precautions in field settings. The sanitary measures described below require some practice in mock situations before their implementation in the field, to allow researchers to become accustomed to appropriate face‐mask wearing and the routine of regular decontamination of any material that could come in contact with a wild mammal.

People suspecting or testing positive for SARS‐CoV‐2 infection should wait until the end of their instructed quarantine period before interacting with mammals. Fieldwork with any mammal should be suspended if the fieldworker is coughing, sneezing or generally feeling ill. However, SARS‐CoV‐2 can be transmissible before the onset of symptoms or in the absence of symptoms. Therefore, similar to precautions taken among people to avoid spreading SARS‐CoV‐2, one should keep physically distant from the study animal if possible, also when asymptomatic. The IUCN Primate Specialist Group recommends at least 7 m distance between humans and great apes (Gilardi et al. 2015). The appropriate distance depends on the behavioural characteristics of the species under observation; for example, could the individual move closer to the person quickly and without warning?

Physical distancing is feasible for many wildlife observational studies, such as monitoring populations by counting individuals from a distance. Even in these situations, if one needs to come closer to surfaces that, within the next hours or days, may be in contact with the study animal, one must wear a face mask appropriately in order to avoid leaving saliva or nasal droplets on those surfaces. Similarly, if one needs to touch surfaces that might be in contact with the animal within the next hours or days, wear clean clothes and decontaminate these surfaces. In particular, face masks and clean gloves must be worn when setting up bait stations for e.g. camera trapping and observational studies, to avoid contamination of the material that mammals will be in contact with. ‘Quarantining’ such equipment and material before use is also possible: prepare the material at least 72 hours before use, so that any virus potentially spilled on the material during preparation work is no longer viable before deployment in the field.

If essential fieldwork requires direct contact or coming closer to live mammals than is considered safe, one should follow strict measures:

- Wear clothes or an overall that have been freshly washed with detergent or not used for 72 hours before wearing. Change into such clean or ‘quarantined’ clothes before every field session.
- Wash hands and face before every field session with soap and water, or decontaminate with a hand sanitiser containing >80% ethanol.
- Decontaminate all material and surfaces that may be in contact with the animal within the next hours or days, or that you leave behind in the field and that an animal may touch. Use an appropriate disinfectant such as >80% ethanol, bleach solution (40 mL household bleach in 1 L of water), or a peroxygen compound such as Virkon.
- Wear an appropriate face mask covering your mouth and nose.
- Wear clean gloves.

These sanitary measures are also advised when handling mammals in wildlife rehabilitation centres. Animals cared for by staff suspecting or testing positive for SARS‐CoV‐2 infection should be isolated from other animals, kept in captivity for at least 14 days, and monitored for clinical symptoms. Samples taken by veterinarians should be tested for SARS‐CoV‐2 infection. Animals testing positive should remain isolated from other animals, and care should be discussed with a veterinarian. The animal needs to test negative twice, five days or more apart, before release.

**Which face masks should be worn, and how should they be handled?**

It is important to keep in mind that the aim is to prevent transmission of one’s own pathogens to wild mammals, rather than the reverse. To avoid spreading our own saliva or nasal droplets, a reusable cloth mask (with at least two layers of fabric) or a disposable surgical mask is appropriate, much like the masks surgeons wear to prevent transmitting their own respiratory microbes to their patients.

Respirator masks, such as FFP2/N95 or FFP3/N99, are more closely sealed and filter finer particles, but are rather meant for protecting oneself from (aerosolised) microorganisms. If such respirator masks must be worn as Personal Protective Equipment (PPE) under particular field work conditions, make sure they do not contain an exhalation valve; such a valve may increase breathing comfort but will allow the outward passage of droplets.

Similarly, most power‐aired purifying respirators (PAPRs), often worn as PPE when handling animals that are potentially infected with very dangerous airborne pathogens, do not protect against spreading an infection. Indeed, in most PAPRs, only the incoming air is filtered, while the outgoing air, containing human respiratory droplets, is not filtered and is even expelled under positive pressure. Therefore, if PAPRs are required for personal protection during field work with mammals that are released alive, make sure that the PAPR‐hood also contains a filter for the outgoing air.

When using a reusable cloth mask, make sure it has been decontaminated (e.g. by washing with detergent) and dried before every field session. After removing and replacing a face mask during a field session (e.g. when taking a break), the inside of the mask should not be handled, as this contains the wearer’s respiratory droplets, and hands should be decontaminated afterwards. If several masks are available per day, it is convenient to wear a new clean one every time a mask is removed.

**Which gloves should be worn, and how should they be handled?**

Clean, disposable latex or nitril gloves should be worn whenever handling an animal or material and surfaces that may be in contact with the animal within the next hours or days, or that you leave behind in the field and that an animal may touch. If gloves of a sturdier material such as kevlar or leather need to be used for safe handling of the animal, larger sized nitril or latex gloves can be worn over them. If this is not possible, make sure that the sturdy gloves remain clean, do not touch your skin or any material that you come in contact with while wearing these gloves, and disinfect them before handling.

When putting on clean gloves, only the sleeve edge of the glove should be touched with bare hands; the wearer should avoid touching the rest of the glove. People wearing gloves should not touch their face or items that may be contaminated with respiratory droplets. If this should happen, they should change the gloves or decontaminate them with a disinfectant.

SARS‐CoV‐2 may also reach wild mammals via non‐human intermediate hosts that people have more frequent contact with, such as pets, livestock, and feral and (semi‐)commensal animals, which, in turn, might contact and infect wild animals. Pets that are infected by their owners, as has been documented on several occasions (Box 1), and that can roam freely outdoors, can transmit the infection to wildlife. While there have been no reported SARS‐CoV‐2 transmissions to livestock such as cattle *Bos taurus*, pigs *Sus scrofa domesticus* or dromedaries, SARS‐CoV‐2 outbreaks in American mink have, to date (20 August 2020), been reported in 42 different mink farms in four countries, suggesting that this species is highly susceptible and easily transmits SARS‐CoV‐2, also via air.

The fact that mink have been reported to transmit the virus to at least two humans and several cats is a stark example of how amplified SARS‐CoV‐2 infection in a highly susceptible and dense animal population could form an efficient transmission bridge between the human SARS‐CoV‐2 reservoir and other susceptible animals (Oreshkova et al. 2020). Populations of stray or feral cats, occurring in high densities in many urban areas around the world (Robertson 2008), could present another situation in which SARS‐CoV‐2 could be amplified. To our knowledge, SARS‐CoV‐2 surveillance of feral cats has not yet been reported. Transmission of other pathogens among domestic, feral, and wild cats, and from domestic cats to other mammal species, has been demonstrated repeatedly (Twardek et al. 2017, Chalkowski et al. 2019), and cats are highly susceptible to SARS‐CoV‐2, possibly via airborne transmission (Bosco‐Lauth et al. 2020, Halfmann et al. 2020, Shi et al. 2020).Structure homology modelling that

Mammals that probably most frequently encounter people are those with a commensal, semi‐commensal, and peri‐domestic lifestyle. Some species, furthermore, thrive in urban environments; in total, those are likely to have the most human‐encounters of the sort that allow transmission of a respiratory virus such as SARS‐CoV‐2. Fortunately, the most common urban dwellers worldwide are *Mus* spp. and *Rattus* spp., which are unlikely to be susceptible to SARS‐CoV‐2 (Table 1). This assumption is based on experimental inoculation of laboratory mice *Mus musculus domesticus* where viral replication failed, and *in vitro* assays that show that SARS‐CoV‐2 cannot enter cells with the ACE2 receptor of *Mus musculus domesticus* or *Rattus norvegicus* (Bao et al. 2020, Zhao et al. 2020b). Structure homology modelling that compares this ACE2 receptor with other members of the Muridae family indicate that SARS‐CoV‐2 probably also cannot enter cells of other murid species (Damas et al. 2020), a number of which have a semi‐commensal or peri‐domestic lifestyle in the Old World. Animal infection experiments have shown that SARS‐CoV‐2 is able to infect Syrian hamsters and the North American deer mouse *Peromyscus maniculatus*, and ACE2 homology modelling showed that SARS‐CoV‐2 is also likely to be able to infect other members of the Cricetidae family (Damas et al. 2020, Griffin et al. 2020, Sia et al. 2020). While rarely fully commensal, many cricetids, especially in rural areas in the Americas, live semi‐commensally and peri‐domestically. Egyptian fruit bats also have demonstrated SARS‐CoV‐2 susceptibility and an occasional peri‐domestic occurrence (Schlottau et al. 2020), but it is unknown whether related fruit bats are also susceptible.

Susceptible animals living in anthropogenic environments, such as feral cats and semi‐commensal small mammals, are thus at relatively high risk of acquiring SARS‐CoV‐2 infection from humans and of further transmitting it to other wild mammals. Unfortunately, it is extremely challenging to devise and implement sanitary measures to prevent the initial transmission events from humans to such potential intermediate hosts. We will therefore have to rely mainly on early and active surveillance of likely intermediate hosts for control of these indirect transmission routes from humans to wildlife.

## WHAT COULD BE THE CONSEQUENCES OF HUMAN‐TO‐WILDLIFE TRANSMISSION OF SARS‐CoV‐2?

### Disease in wild mammals

In humans, COVID‐19 symptoms range very widely, including mild respiratory problems, gastro‐intestinal issues, headaches, severe acute respiratory syndrome, and no symptoms at all (Guan et al. 2020, Pan et al. 2020). The infection mortality rate of COVID‐19 in humans appears to be around 0.7%, but varies widely with age and co‐morbidities (Verity et al. 2020). This percentage could still change with better knowledge of numbers of undocumented infections. The few examples of natural and experimental infections in macaques, hamsters, cats, tigers, and lions suggest that they experience similar symptoms as in mild or moderate human cases, but pneumonia and an increased mortality was noted during SARS‐CoV‐2 outbreaks in some but not all mink farms (Molenaar et al. 2020). If severe pathogenicity had occurred frequently in pets such as cats and dogs, we assume this would have been noticed already, suggesting at most a relatively mild disease in these species. The human coronavirus HCoV‐OC43, which causes mild respiratory symptoms in humans, also caused mild respiratory symptoms in a population of habituated chimpanzees *Pan troglodytes* when this infection was introduced to them via humans (Patrono et al. 2018). Other mild human respiratory viruses transmitted to great apes have, however, led to severe disease and mortality (Köndgen et al. 2008). An alpha CoV that commonly circulates in domestic cats spread efficiently among all 60 cheetahs *Acinonyx jubatus* in a safari park, causing severe disease and a high mortality rate in the cheetahs, while infected lions did not show any overt symptoms (Wilkerson et al. 2004).

Not only direct mortality or severe disease is a concern. Wild mammals often live on the edge of survival, so even a mild disease may result in lower survival or reproduction probabilities. Natural stressful situations, such as food shortages and co‐infections, may also pre‐dispose wild mammals to a more severe disease. Infection with equine coronavirus has a low mortality rate among domestic horses *Equus ferus caballus*, but causes clinical problems such as leukopenia and metabolic disturbances that can have serious secondary consequences in wild mammals (Berryhill et al. 2019). Besides the severe, often lethal diarrhoea that it causes, porcine epidemic diarrhoea virus also considerably reduces the reproductive performance of sows (Furutani et al. 2018). Even without apparent disease symptoms, virus infections may have important fitness consequences: although Puumala hantavirus does not cause clinical illness in its natural host the bank vole *Myodes glareolus*, winter survival of infected animals is affected and there are age‐dependent effects on reproduction (Kallio et al. 2007, Kallio et al. 2015).

It is difficult to predict the pathogenicity of SARS‐CoV‐2 in new mammalian hosts in the wild. As evidence is lacking for the absence of morbidity or mortality for most mammalian species, great caution is needed, especially to prevent SARS‐CoV‐2 transmission to threatened species.

### Establishment of a novel reservoir

As several mammal species are susceptible to SARS‐CoV‐2 and the virus appears to be transmitted easily among humans and experimentally infected susceptible mammals (see Box 1), SARS‐CoV‐2 has the potential to spread very quickly in a wild mammal community. If the virus can circulate uninterruptedly for some time, eventually a new non‐human reservoir could be established. This scenario would pose a significant hurdle for efforts to control SARS‐CoV‐2 in the human population. The new, much less controllable, wildlife source would have the potential to start new epidemics in humans, even when transmission among humans had been stopped in an area. It may also provide new opportunities for evolutionary changes in the virus, with potential consequences for transmissibility and pathogenicity in humans and effectiveness of a vaccine.

Establishment of a SARS‐CoV‐2 reservoir in wildlife populations could furthermore lead to negative and even hostile perceptions of these species among humans. Bats already have a bad connotation in different parts of the world, and have suffered mass cullings in reaction to their association with disease (Kingston 2016). This aversion appears to be increasing, since bats have been mentioned as the probable source of the present pandemic in the media. Even in China, where bats are traditionally symbols of good luck and happiness, they are more at risk since the COVID‐19 pandemic (Zhao 2020). The known presence of a virus dangerous to humans in particular mammals could severely complicate field research on these species and tourism activities, both often greatly needed for their conservation.

The likelihood of SARS‐CoV‐2 successfully spreading in a wild mammal population after starting from, for example, a single infected animal depends on for how long the individual remains infectious and on the population‐ecological and behavioural characteristics that determine the contact frequency between animals. For example, the infection is less likely to spread among mammals with solitary lifestyles than among mammals that live in herds or large family groups. The sustainability of transmission chains in the long term further depends on the overall abundance of susceptible animals. The latter in turn depends on the population size, the turnover rate in the population due to births of new susceptible individuals, the proportion of individuals that are already immune (and the duration of that immunity), the probability that any co‐occurring related CoVs induce cross‐immunity, and the connectivity between (meta‐)populations (Anderson et al. 1992, Keeling & Grenfell 1997, 2000, Reijniers et al. 2012). For a virus like SARS‐CoV‐2 that can infect a wide range of mammal species, it is relevant that connected meta‐populations can extend across different species in the community. This may increase the abundance of susceptible individuals in comparison with a single species, thus increasing the probability of long‐term persistence of SARS‐CoV‐2 transmission. Furthermore, through such inter‐species connections in direct or indirect contact networks, the infection could (repeatedly) reach individuals of species of which population densities and contact frequencies would be too low to maintain transmission. As such, a cascade of transmission chains among interconnected populations of different SARS‐CoV‐2‐susceptible species could ultimately also reach threatened species. A similar phenomenon is documented for the plague *Yersinia pestis* in black‐tailed prairie dog *Cynomys ludovicianus* colonies in the USA. The disease repeatedly reaches and kills the Critically Endangered black‐footed ferret *Mustela nigripes* (Matchett et al. 2010). In fact, plague is an excellent example of how a pathogen can act as an invasive transformer species affecting the stability of the ecosystem (Eads & Biggins 2015).

With the many variables involved, it is difficult to quantify the likelihood that one individual wild mammal infected by a human or an intermediate host would result in the infection continuing to spread among populations and establishing a new SARS‐CoV‐2 reservoir. From experience of infections emerging in human populations, we can qualitatively assess that the risk is actually rather low and prone to stochasticity. Humans have frequent interactions with wild mammals through hunting, the wildlife meat trade, markets with (live) wildlife, and occupational activities (e.g. bat guano harvesters, forestry workers, and wildlife researchers). Humans themselves have a unique global contact network containing well‐connected high‐density clusters. Still, the viral infections that humans probably often acquire from wildlife only very rarely lead to long human‐to‐human transmission chains (Woolhouse et al. 2016). Most wild mammal populations or even multi‐species communities are perhaps less suited for sustained SARS‐CoV‐2 transmission than human populations. On the other hand, the number of contacts from the same human reservoir to a wild mammal species may be higher than in the reverse situation, given the immensely large reservoir of millions of SARS‐CoV‐2 infected people globally. The larger the proportion and absolute number of infected people, the higher the probability that at least one of these contacts would lead to successful onwards transmission in wild mammals. In any case, even if the risk is low, the stakes of sustained SARS‐CoV‐2 transmission in wildlife are very high. The relatively simple precautions outlined in Box 2 can substantially reduce the likelihood of fieldworkers transmitting the infection to wild mammals.

## CONCLUSION

The likelihood that mammologists, conservationists, wildlife field researchers, or other people interacting directly or indirectly with wild mammals initiate a chain of SARS‐CoV‐2 transmission among wild mammal populations is not negligible, and is probably higher than with other common human viruses. Indeed, SARS‐CoV‐2 presents unique features: it is currently present in a significant proportion of humans worldwide; it is highly transmissible through direct and indirect contact and via airborne droplets; many people are infectious before or without symptoms (so that quarantining only when sick is not sufficient to prevent transmission); and finally, the virus is able to infect a wide range of distantly related mammals, and predicting which animal species are susceptible seems challenging. Combined with the potential devastating impact on both humans and wild mammalian populations that sustained SARS‐CoV‐2 transmission in wildlife would have, we urge people to take sensible sanitary precautions when in contact with any wild mammal species, in order to reduce the risk of human‐to‐wildlife SARS‐CoV‐2 transmission as much as possible. Indirect transmission routes from humans to wildlife via domestic or feral mammals acting as intermediate hosts are more difficult to prevent. Controlling these routes will therefore depend on early and active surveillance of domestic and feral mammals for signs of SARS‐CoV‐2, which we also recommend.
